# Mandibular asymmetry: a three-dimensional quantification of bilateral condyles

**DOI:** 10.1186/1746-160X-9-42

**Published:** 2013-12-20

**Authors:** Han Lin, Ping Zhu, Yi Lin, Shuangquan Wan, Xin Shu, Yue Xu, Youhua Zheng

**Affiliations:** 1 Department of Orthodontics, Guanghua School of Stomatology, Hospital of Stomatology, Sun Yat-sen University, Guangdong Provincial Key Laboratory of Stomatology, Lingyuan Xilu No.56, Guangzhou 510055, China

**Keywords:** Temporomandibular joint, Mandibular asymmetry, Condylar morphology, Curvature analysis, Computed tomography

## Abstract

**Introduction:**

The shape and volume of the condyle is considered to play an important role in the pathogenesis of the mandibular deviation. Curvature analysis is informative for objectively assess whether the shape of the condyles matches that of the glenoid fossa. In this study, a three-dimensional (3-D) quantification of bilateral asymmetrical condyles was firstly conducted to identify the specific role of 3-D condylar configuration for mandibular asymmetry.

**Methods:**

55 adult patients, 26 males (26 ± 5 yrs) and 29 females (26 ± 5 yrs), diagnosed with mandibular asymmetry were included. The examination of deviation of chin point, deviation of dental midlines, inclination of occlusal plane, and depth of the mandibular occlusal plane were conducted. After the clinical investigation, computed tomography images from the patients were used to reconstruct the 3-D mandibular models. Then the condylar volume, surface size, surface curvature and bone mineral density were evaluated independently for each patient on non-deviated and deviated sides of temporomandibular joint.

**Results:**

Both the condylar surface size and volume were significantly larger on deviated side (surface size: 1666.14 ± 318.3 mm^2^, volume: 1981.5 ± 418.3 mm^3^). The anterior slope of the condyle was flatter (0.12 ± 0.06) and the posterior slope (0.39 ± 0.08) was prominently convex on the deviated side. The corresponding bone mineral density values were 523.01 ±118.1 HU and 549.07 ±120. 6 HU on anterior and posterior slopes.

**Conclusions:**

The incongruence presented on the deviated side resulted in a reduction in contact areas and, thus, an increase in contact stresses and changes of bone density. All aforementioned results suggest that the difference existing between deviated and non-deviated condyles correlates with facial asymmetrical development. In mandibular asymmetry patients, the 3-D morphology of condyle on deviated side differ from the non-deviated side, which indicates the association between asymmetrical jaw function and joint remodeling.

## Introduction

Mandibular deviation is one of the common craniofacial deformities with a lateral shift in the midline of the mandible [1], which results from the asymmetric growth of mandible or other certain diseases affecting the facial growth. On the other hand, imbalanced occlusion in patients with mandibular asymmetry can cause abnormal stress distribution on articular surfaces and dysfunctional osseous remodeling of condyles, causing the internal derangement and functional impairment of the temporomandibular joints (TMJs) and finally leading to osteoarthritis [2-4].

The condyle plays an important role as the primary center of growth in the mandible and serves as the pivot end of the jaw rotating in the skull. Its surface morphology and bone density correlate with the pathogenesis of mandibular asymmetry and bilateral imbalanced occlusal force. With advances in anthropometry techniques, various studies attempt to objectively quantify maxillofacial tissue asymmetry with computed tomography (CT) [5], magnetic resonance image (MRI) [6], and cephalometric analyses [7]. However, most of the reports are limited to the linear measurement of condyle, such as lengths, angles and vectors. Saccucci *et al.*[8] demonstrated that the optimum size or volume of the mandibular condyle are indicative and predictive of a precise clinical situation. Previous researches emphasized much on the overall condyle, while the condylar surface characteristics are overlooked [9,10]. However, the contour changes of articular cartilage and subchondral bone, rather than the overall shape modification of joint, may be the initial manifestation of morphologic alterations on articular surfaces.

TMJ, a “loose-fitting”, rotating and sliding joint, moves like a door hinge. Its unique structure and complex function contribute to the distinguished pattern of stress distribution in the joint [11,12]. The surface configuration (e.g. condyle curvature, superficial area, and volume) and the properties of subchondral bone (e.g. trabecular distribution and bone mineral density) indicate the mechanical stress exerted on the condylar surfaces. Previous studies have suggested that temporomandibular disorders caused by imbalanced occlusal force are embodied chiefly in reconstruction disequilibrium between subchondral osteoblast and osteoclast [13]. While other authors deem temporomandibular disorders (TMDs) as an autoimmune degenerative disease owing to subtle lesions of synovial membrane and cartilage tissue, which may cause the changes of condylar configurations [14,15]. Therefore, 3-dimensional (3-D) reconstruction with CBCT can provide more information about the configuration and bone changes of condyles other than simple distances and angles measurement reported by the previous researchers [16,17].

In order to detect the subtle differences in patients with mandibular deviation, the volume, surface size, curvature and bone mineral density (BMD) of 3-D constructed condylar models on both non-deviated and deviated sides were measured in these patients, whose clinical examinations were collected. The results were therefore compared with the aim of providing a more objective quantification way for evaluating the facial asymmetry and more useful information for the understanding in etiology or symptom of asymmetric mandible.

## Materials and methods

### Ethics statement

The study protocol of retrospective review was approved by the institutional review board at the Sun Yat-sen University. All patients in the study group had consented to be a part of trial after clinical briefing on methodology, and they were informed of the details of the study with signed informed consents signed. The reconstruction models were approved by the institutional ethics board of the Hospital of Stomatology, Sun Yat-sen University. This study is a part of our serial studies of temporomandibular disorders, which has been registered on *Clinicaltrials.gov* (Identifier: NCT00932594).

### Patient population

According to the preliminary experiment and the formula of samples size calculation of paired design and the formula of samples size calculation of group design (alpha value = 0.05, and the statistical power = 0.9), the sample size was finally decided of 55.

55 young adult patients with clinically proven mandibular asymmetry and with available 3-D cone beam CT scan images from September 2010 to May 2013 were included in the study. 26 males (26 ± 5 yrs) and 29 females (26 ± 5 yrs) were retrospectively analyzed and retrieved from the computer data base. Exclusion criteria were proven mandibular fracture, previous mandibular surgery and trauma. The non-deviated side is defined as the shortened side, and the deviated side is the lengthened side of mandible.

### Clinical examination

The following clinical investigations for each patient were collected:

1. deviation of chin point,

2. deviation of dental midlines,

3. inclination of occlusal plane, and

4. depth of the mandibular occlusal plane.

1. Deviation of chin point

Deviations of the chin point was measured as the distance between the chin point and the facial midline directly on the patients. The facial midline was defined as the perpendicular bisector of the line between the centers of the right and the left pupils.

2. Deviation of dental midlines

Deviation of dental midlines was defined as the horizontal distance between mesial contact points of maxillary central incisors and mandibular central incisors, measured directly on the patients.

3. Inclination of occlusal plane

Patients were asked to bite on a tongue blade, and then the cant in occlusal plane was detected with the angle between the blade and the iner-pupillary plane.

4. Depth of the mandibular occlusal plane

The vertical distance from the mandibular occlusal plane to the lowest cusp in mandible was measured both on the deviated and the non-deviated side. First, a flat plane is laid on top of the mandibular dental cast touching the incisal edges of the central incisors and the distal cusp tips of the most posterior teeth in the lower arch. Then, the perpendicular distance between the deepest cusp tip and the flat plane is measured. The measurement was made on the right and left side of the dental arch.

### 3-D measurement of condyles

Non-deviated (shortened side) and deviated sides (lengthened side) of TMJs were evaluated independently for each patient. TMJ evaluation included:

1. Condylar volume and surface size,

2. Condylar surface curvature analysis, and

3. Condylar BMD measurement.

### The condylar volume and surface size calculation

CBCT data sets were acquired with a DCT Pro CBCT (Vatech, Co., Ltd., Hwasung, Korea) using the following scanning parameters: 90 kVp, 24 s, 4 mA, voxel size 0.4 mm and field of view 20 × 19 cm. The image covered the area from the upper orbits rim to the inferior border of the mandibular body. The gross data and the slices obtained were imported and reconstructed into 3D models by an interactive image system (Materialise’s interactive medical image control system, Mimics, 15.0; Materialise, Leuven, Belgium).

**Figure 1:**
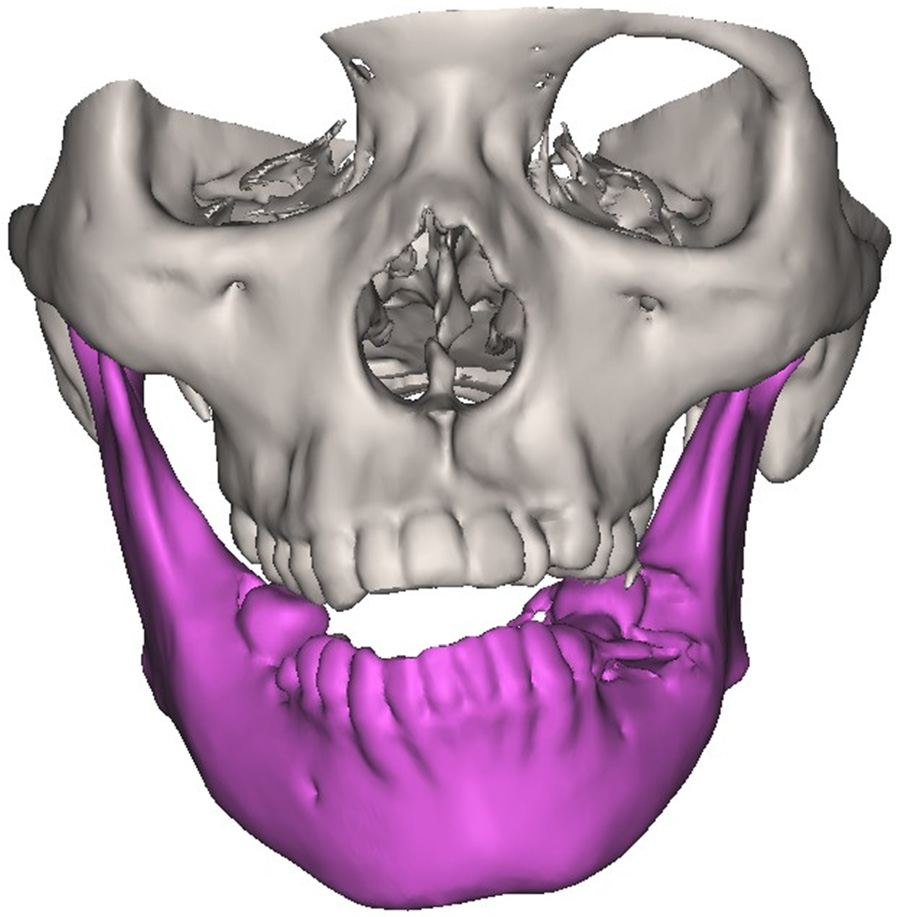
**The 3-D constructed model of a mandibular asymmetry patient.** The purple part indicated the mandibular model.

**Figure 2:**
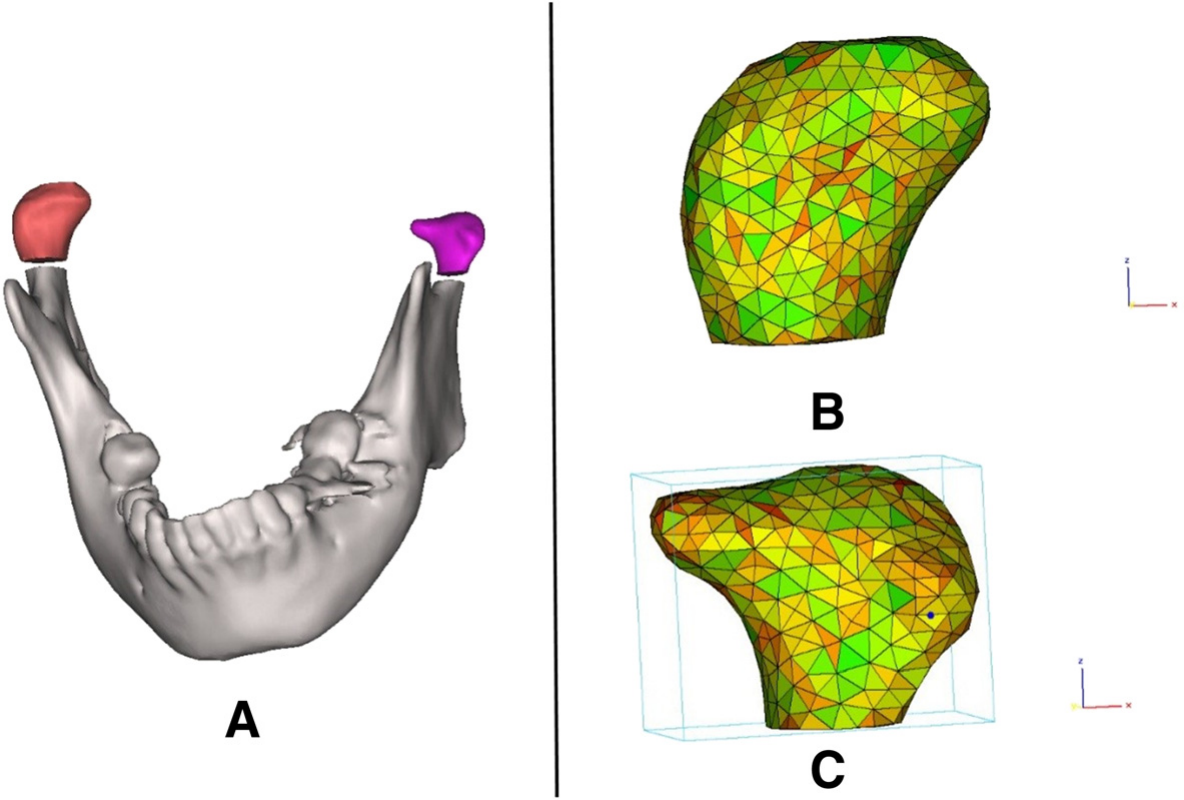
**The 3-D constructed model of mandibular.** On the constructed mandible **(A)**, condyles of non-deviated **(B)** and deviated **(C)** sides were separated from the mandible and were mashed further.

The condyle was visualized in the recommended bone density range (range of HU from 226 to 3071) isolated prior to making 3-D measurements. Then, the TMJ was separated from the 3-D model (Figure 1). The condyle CT data set were further segmented with a dedicated Mimics^TM^ tool to construct a mask, which included only the mandibular condyle. After the isolation, three-dimensional multi-planar reconstructions were performed for each condyle using a Mimics tool (Figure 2). The upper and lower limits of condyle were defined according to Tecco *et al*. [18] volumetric (mm^3^) and surface size measurements (mm^2^) were made for condyles on two sides through the Mimics™ automatic function.

### Condylar surface curvature analysis

In Mimics software, the contours of condyle surfaces were extracted and reconstructed into polygon-based models. Triangulation between contiguous slices was performed based on a Euclidean distance measure. Each contour point in the next slice to form a triangle. The models were then export as stl^*^ files. The models exported as stl^*^ files were then imported into Rhinoceros 4.0 software (Robert McNeel & Associates, Seattle, USA) for Gaussian Curvature Analysis to gain information about the type and amount of curvature on the surface. Gaussian curvature is one of the most essential geometric invariants for surfaces. Different sections will have different curvatures, the maximum and minimum values of these are called the principal curvatures, called κ1and κ2. The Gaussian curvature is the product of the two principal curvatures Κ = κ1× κ2. It is an intrinsic measure of curvature. The sign of the Gaussian curvature can be used to characterize the surface. Κ equal to, more than, or less than zero stands for flat, spherical or hyperbolic shape respectively.

The articulating surface of the condyle extends both anteriorly and posteriorly to the most superior aspect of the condyle. From the superior view, the anterior and posterior slopes are divided by the transverse ridge. The values of mean Gaussian curvature on the anterior and posterior slopes of condyles were measured and compared. The condyles were plotted as different colors, revealing the curvature characteristic visually.

### Condylar BMD measurement

**Figure 3:**
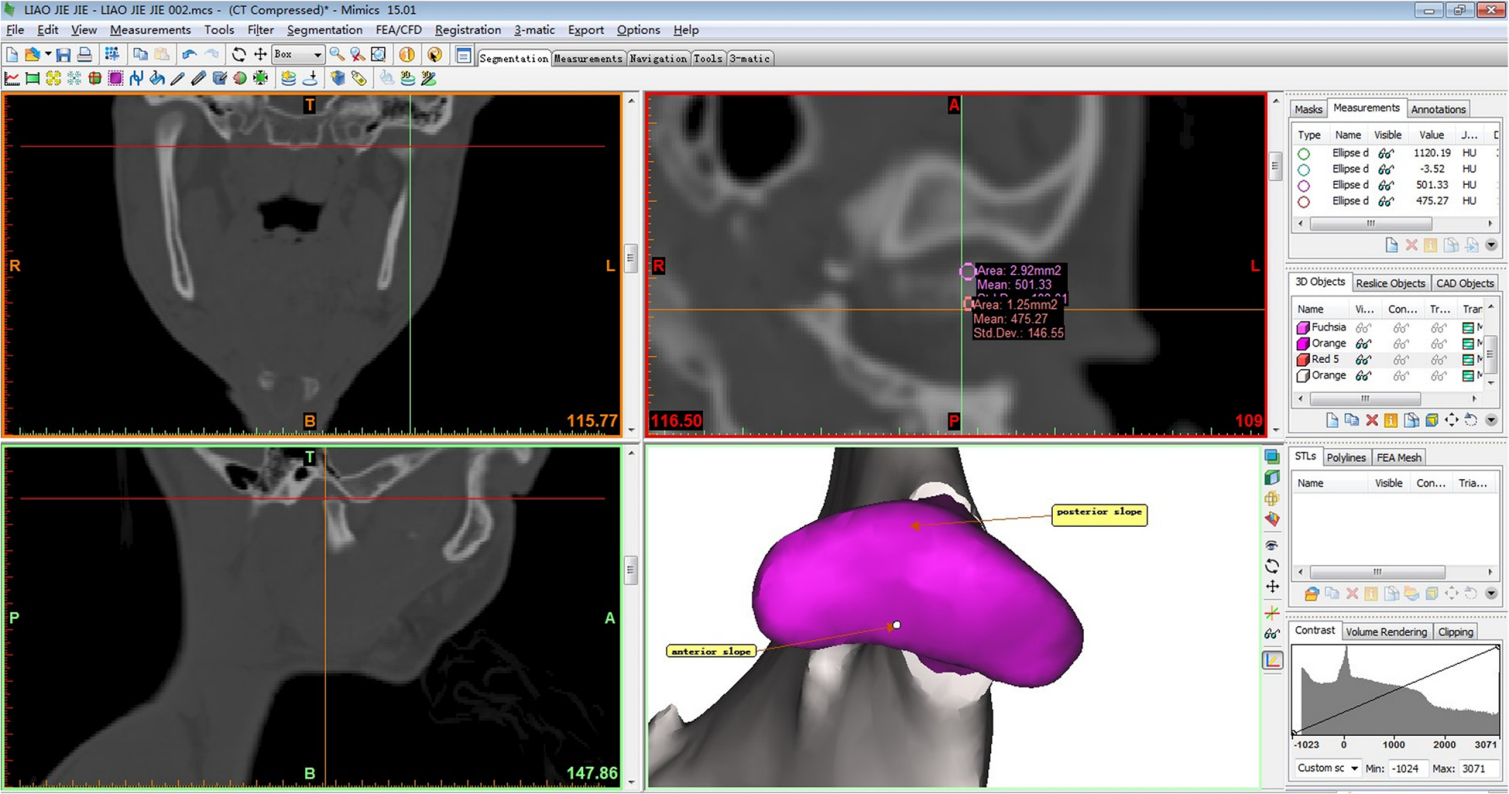
**Bone mineral density (BMD) measurement of anterior and posterior slope of condyle.** HU measurement made in CT image using MIMICS software.

Hounsfield Unit (HU) was measured in the regions of the anterior and posterior slope of condyles (Figure 3). The mean HU value was substituted in the following equation to estimate apparent physical density of the bone (Est-vol. BMD, g · cm^-3^). Est-vol. BMD (g · cm^-3^) = 0.114 + 0.916 × 10^-3^ (HU) [19].

In the paper, the mean HU values were used directly for comparison of deviated and non-deviated sides of condyles. A constant ellipse area of 2.92 mm^2^ was used for the measurement.

### Statistical analysis

The data, presented as mean ± standard deviation, were processed and analyzed using SPSS 16. 0 (SPSS Inc, Rainbow Technologies, Chicago). All data were normally distributed. The paired sample *t*-test were used to calculate the statistically significant differences of volume and surface size between the non-deviated and deviated sides. Bonferroni method was used for comparison of mean Gaussian curvature and BMD at anterior and posterior slope of the both condyles. An ANOVA of Randomized Complete Block-design was performed to do the analyses of 2 sides and 2 parts of each side. In our preliminary experiment, the consistency of the measurement method was assessed by one-way random intra-class correlation coefficients (ICCs), the ICCs were all above 0.9, showing the high reliability of these measurements.

## Results

The results of clinical examination were shown on Table 1. Significant difference (*P* < 0.05) was found between the depth of the mandibular occlusal plane on non-deviated and deviated side.

**Table 1:** The mean values of clinical examination

**Variable**	**Mean ± SD**
Deviation of extra-oral midline (mm)	5.2 ± 2.0
Deviation of dental midlines (mm)	3.1 ± 1.2
Inclination of occlusal plane (°)	4.6 ± 2.4
Depth of the mandibular occlusal plane:	
Non-deviated side (mm)	2.0 ± 0.5
Deviated side (mm)	1.5 ± 0.8

**Figure 4:**
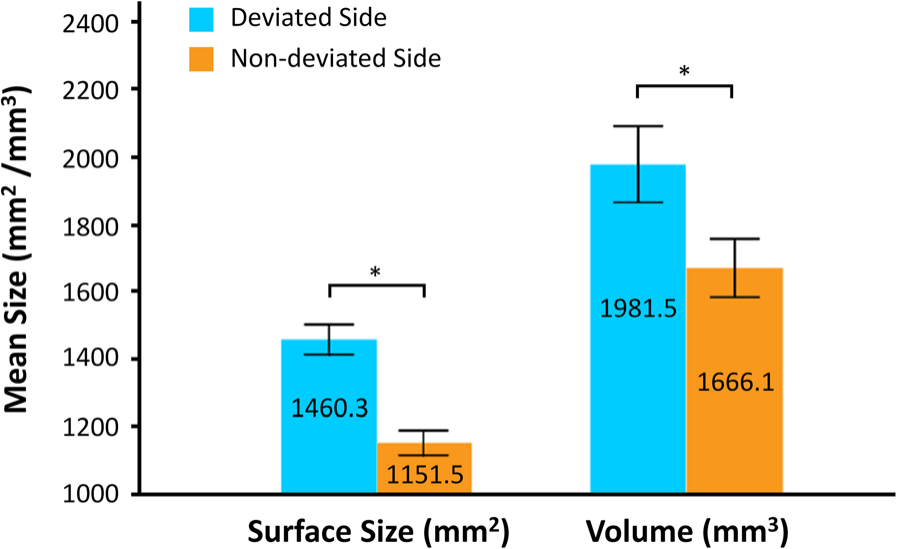
The surface size and volume of deviated and non-deviated condyles (*P **< 0.001).**

The condylar volume was 1981.5 ± 418.3 mm^3^ on deviated side and 1460.3 ± 165.0 mm^3^ on non-deviated side, with significant difference (*P* < 0.001). The same was observed for the condylar surface size (1666.14 ± 318.3 mm^2^ on deviated side and 1151.5 ± 134.7 mm^2^ on non-deviated side, *P* < 0.001) (Figure 4).

**Figure 5:**
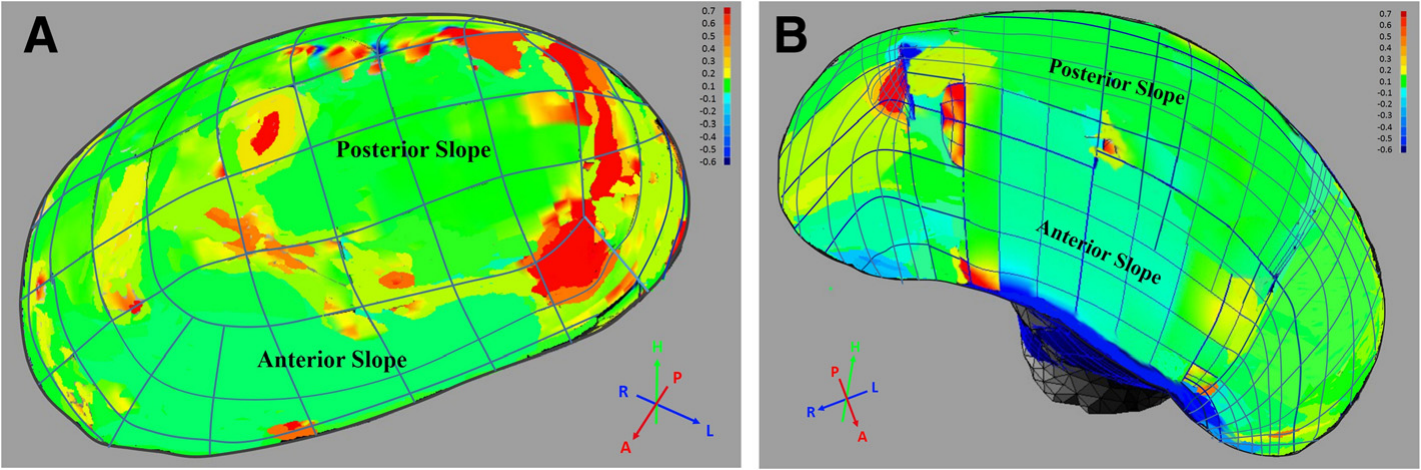
**The color maps of curvature analysis.** The anterior and posterior slopes were labeled both on deviated **(A)** and non-deviated **(B)** sides.

**Figure 6:**
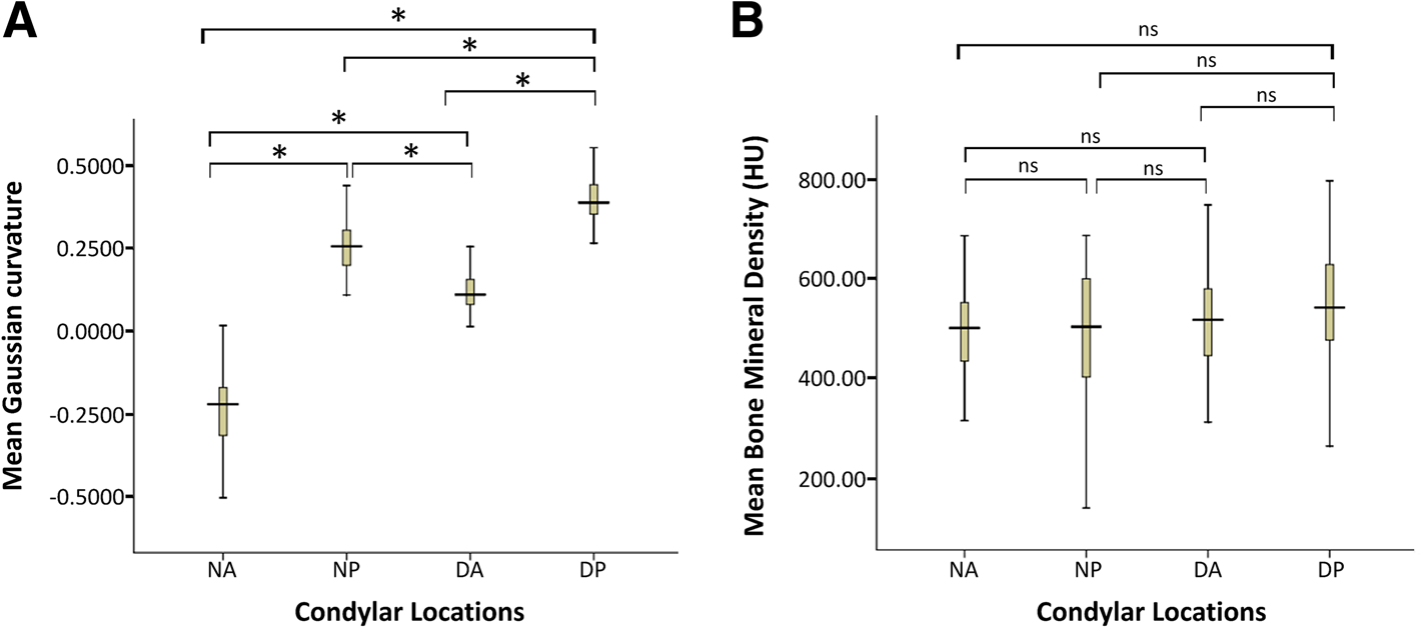
**Comparison of mean Gaussian curvature and BMD.** Comparison of mean Gaussian curvature **(A)** with significant difference (*P* < 0.001) between groups, and mean BMD **(B)** without statistical difference (*P* > 0.05) between groups. The comparison was performed on the anterior and posterior slopes of bilateral condyles. “N” and “D” represent the non-deviated and deviated condyles, while “A” and “P” represent the anterior and posterior slopes respectively. (*P < 0.001; ns, not significant).

The local curvature values were plotted as color maps onto 3D-reconstructions of condyle surfaces of the deviated and non-deviated sides (Figure 5). The deviated condyle has flatter surface, while the non-deviated condyle has more concave-convex surface. The mean Gaussian curvature of anterior slope of condyle on deviated side (0.12 ± 0.06) was higher than non-deviated side (-0.23 ±0.11). Significant difference was observed on the posterior slope of condyles (0.39 ± 0.08 on deviated side and 0.26 ± 0.07 on non-deviated side, *P* < 0.001). The anterior slope of condyle on deviated side had the smallest value of mean Gaussian curvature of negative sign (Figure 6A).

BMD was 497.02 ±116.2 HU and 485.30 ±148.4 HU on anterior and posterior slope of non-deviated side respectively. On deviated side, the corresponding values were 523.01 ±118.1 HU and 549.07 ±120. 6 HU. No statistically significant difference was found between the four groups (Figure 6B).

## Discussions

As the primary center of mandible growth, the condyle undergoes a remodeling process as the responses to continuous stimuli during jaw movements. But asymmetrical jaw function alters the intra-articular mechanical dynamics, which gives rise to persistent or renewed activity in one or both of the condyles [20]. Numerous studies have attempted to evaluate the morphology of the human condyle [21,22]. However, a simple gather of linear distance, angles and vectors is not sufficient to depict the articular surface characteristics. Analyzing on the reconstructed CT models, we quantified the condylar surface size, volume, curvature and BMD on both the deviated and non-deviated sides to provide a more objective quantification way for evaluating the facial asymmetry and more useful information for the understanding in etiology or symptom of asymmetric mandible.

In the current study, 55 patients, whose chin point deviating from the facial midline 5.2 ± 2.0 mm and whose upper and lower dental midline differing 3.1 ± 1.2 mm, were retrospectively reviewed. Then the surface size and volume were calculated by numerically integrating the size of all triangles attributed to the condyle based on the 3-D reconstructed separated TMJ (Figure 1). Both the surface size and volume were significantly bigger on deviated side than on the non-deviated side, consistent with the variety law that the deviated condyle is larger than the non-deviated one found by the previous researchers [23,24]. Habib *et al*. [25] and Kurita *et al*. [26] have shown that movement and function changes of the joint can in turn affect the volume of the condylar head. Quantitative assessment of the joint morphology can be useful for estimating bone loss and hyperplasia in joint disorders. Animal models and epidemiological studies have established that joint size is a relevant risk factor for degenerative joint diseases using MRI data and digital post-processing [4,26,27]. Due to the limitation of detecting bony tissues by MRI technique, these radiological analyses lack detailed information about the condylar surface morphology.

As described by Ueda *et al.*[28], the condylar surface curvature, as a predictor of TMJ congruity, will also influence the masticatory function. In this context, we performed the first 3-D curvature analysis of TMJ, informative for objectively assess the difference of condylar surface curvature between the deviated and non-deviated side. Condyle on the non-deviated side with negative curvature of -0.23 ±0.11 on the concave anterior slope and with 0.26 ± 0.07 on the convex posterior slope, similar to a normal joint shape [3]. While on the deviated side, the anterior slope is flatter (0.12 ± 0.06) and the posterior slope (0.39 ± 0.08) is prominently convex. Hohe *et al.*[29] and Matsumoto *et al.*[30] suggest that joint derangement may occur when the proper morphological adaptation of the articular fossa and condyle is lost. The incongruence presented on the deviated side, predicted by the flatter curvature, results in a reduction in contact areas and, thus, an increase in contact stresses under unchanged applied loads, believed to be responsible for the development of focal lesions in TMJ. Unbalanced stress on TMJs, suggested by Ueki *et al.*[31], is associated with the occlusal plane. When the occlusal plane is inclined, teeth positions are affected and the bilateral occlusal force can be influenced, leading to the unbalanced stress on bilateral condyles [3]. The inclination of occlusal plane in our study is 4.6 ± 2.4 degree, which could induce TMD due to disturbances in stress on the TMJ.

Though no significant difference was detected between the two sides, relative higher BMD was obtained on the deviated side, which has an abnormal surface curvature. The mechanical effects of loading as the initial factor in bone remodeling not only produce alterations in the contour and shape of the subchondral bone but also affect the bone mass [32]. The lower bone density of anterior slope compared to the posterior one may be explained by its role as main burdened surface during the jaw movement. In the current study, significant difference between bilateral depths of the mandibular occlusal plane is shown. The flatter occlusal plane on deviated side resulting in more molar contacts during normal joint movement would increase the forces applied to the TMJs during normal functions [33]. Hence the minor trauma of the anterior slope on the deviated side always acts as the initial sign of TMDs. And a well-balanced position of the condyle relative to the glenoid fossa may be critical to the ordinate function of the TMJ, which should be implicated in the teeth alignment, occlusal treatment and orthotherapy.

## Conclusions

In mandibular asymmetry patients, the 3-D morphology and bone density of condyle on deviated side differ from the non-deviated side, which indicates the association between asymmetrical jaw function and joint remodeling.

## Competing interests

The authors declare that they have no competing interests.

## Authors’ contributions

HL, PZ and YL are the Principal Investigator of this research article. They 1) have made substantial contributions to conception and design, acquisition of data, analysis and interpretation of data; 2) have been involved in drafting the manuscript or revising it critically for important intellectual content; and 3) have given final approval of the version to be published. SW, XS, YX and YZ have made substantial contributions in acquisition of data and participated in drafting the manuscript and helped in the revision of the manuscript. All authors read and approved the final manuscript.
